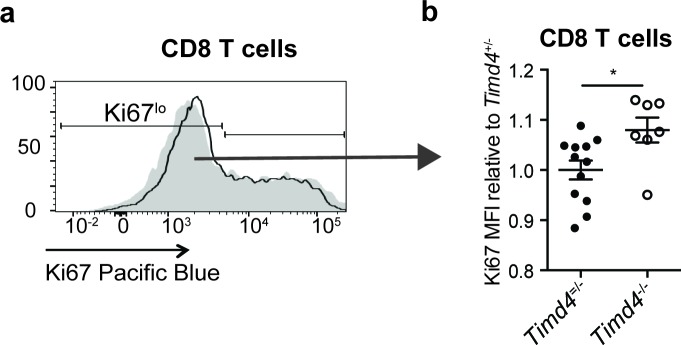# Correction: A role for phagocytosis in inducing cell death during thymocyte negative selection

**DOI:** 10.7554/eLife.56027

**Published:** 2020-02-14

**Authors:** Nadia S Kurd, Lydia K Lutes, Jaewon Yoon, Shiao Wei Chan, Ivan L Dzhagalov, Ashley R Hoover, Ellen A Robey

Kurd NS, Lutes LK, Yoon J, Chan SW, Dzhagalov IL, Hoover AR, Robey EA. 2019. A role for phagocytosis in inducing cell death during thymocyte negative selection. *eLife* **8**:e48097. doi: 10.7554/eLife.48097.

Published 23, December 2019

In the original published version of the article, Figure 3b mistakenly showed the same graph as represented in Figure 4b, which shows CD69 induction on *Timd4*^-/-^ RIPmOVA thymic slices. We had intended to show CD69 induction on RIPmOVA thymic slices treated with Annexin V in Figure 3b, which are the data described in the results as well as the figure legend, and these data are shown in the corrected version.

The corrected Figure 3 is shown here:

**Figure 1:**
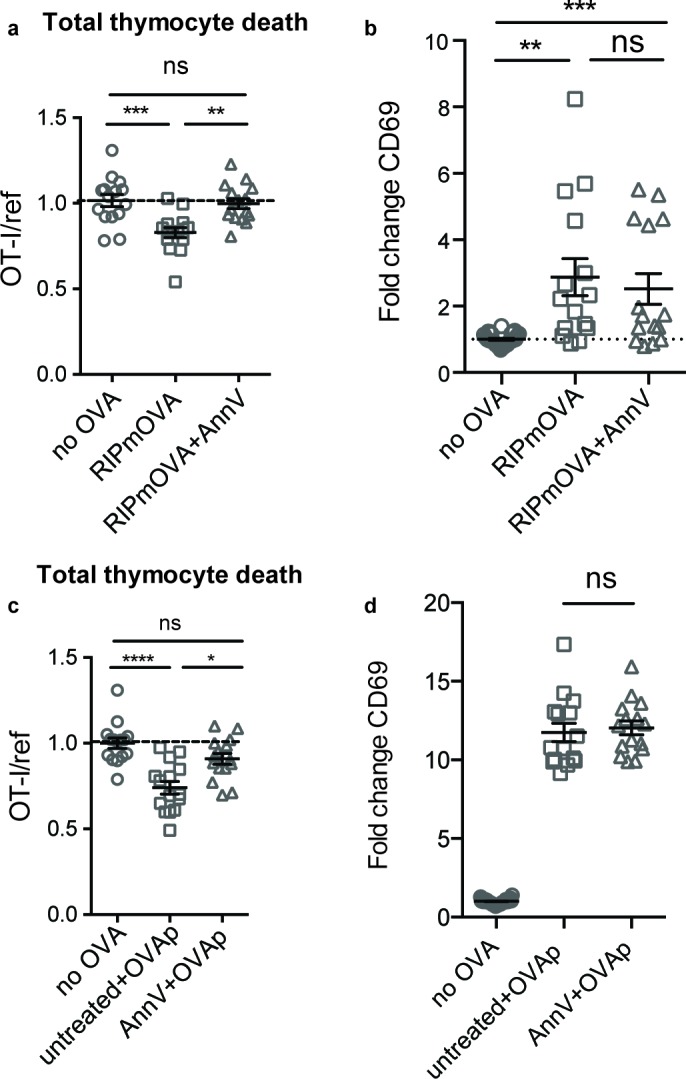


The originally published Figure 3 is also shown for reference:

**Figure 2:**
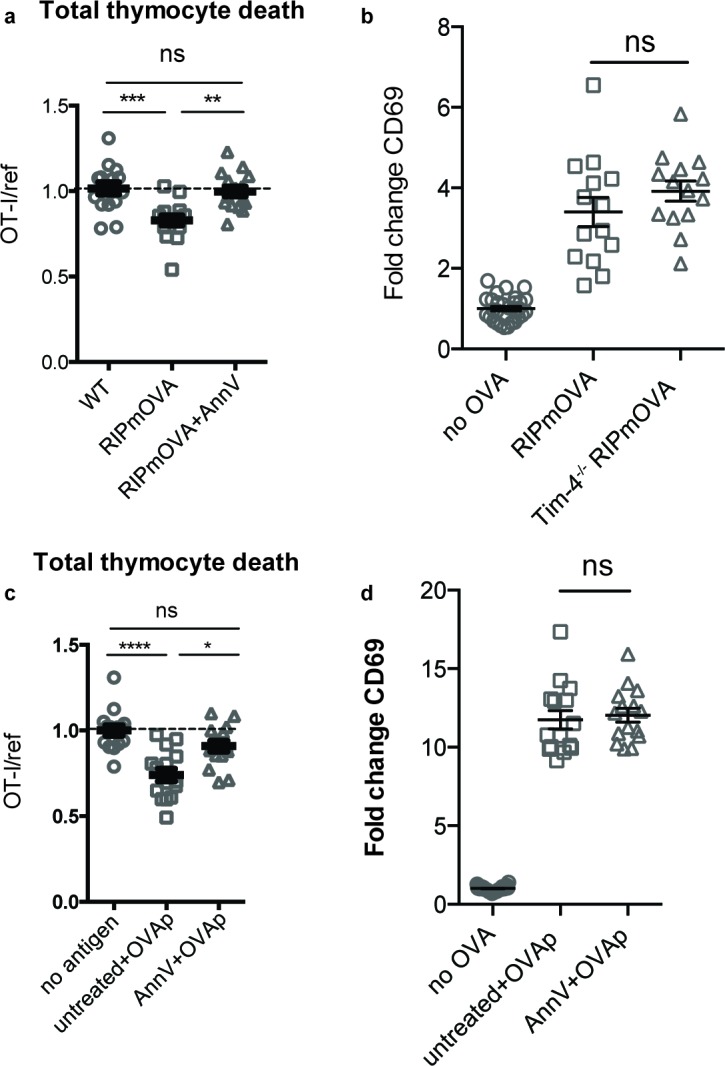


In addition, in Figure 5-figure supplement 1b of the original published version, both columns of the graph are labelled *Timd4*^-/-^. The left column should be labelled *Timd4*^+/-^. The article has been corrected accordingly.

The corrected Figure 5-figure supplement 1 is shown here:

**Figure 3:**
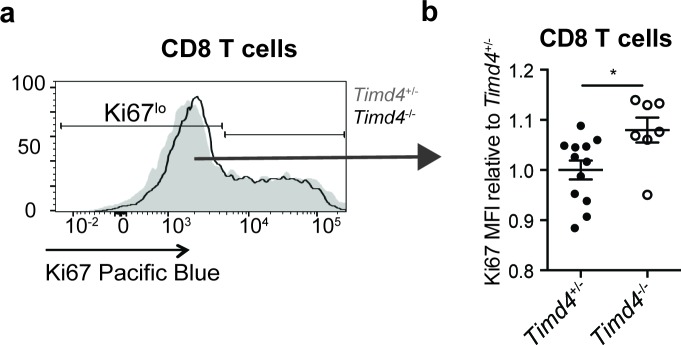


The originally published Figure 5-figure supplement 1 is also shown for reference:

**Figure 4:**